# Gut Microbiota, in the Halfway between Nutrition and Lung Function

**DOI:** 10.3390/nu13051716

**Published:** 2021-05-19

**Authors:** Christophe Espírito Santo, Catarina Caseiro, Maria João Martins, Rosário Monteiro, Inês Brandão

**Affiliations:** 1 Centro de Apoio Tecnológico Agro Alimentar (CATAA) de Castelo Branco, 6000-459 Castelo Branco, Portugal; cespiritosanto@cataa.pt; 2 Centre for Functional Ecology, University of Coimbra, 3000-456 Coimbra, Portugal; 3 Faculdade de Medicina Veterinária, Universidade de Lisboa, 1300-477 Lisboa, Portugal; ccaseiro@fmv.ulisboa.pt; 4 Instituto de Investigação e Inovação em Saúde (i3S), Universidade do Porto, 4200-135 Porto, Portugal; mmartins@med.up.pt; 5 Department of Biomedicine, Unit of Biochemistry, Faculty of Medicine, University of Porto, 4200-319 Porto, Portugal; 6 MEDCIDS-Department of Community Medicine, Information and Health Decision Sciences, Faculty of Medicine, University of Porto, 4200-319 Porto, Portugal; rosariom@med.up.pt; 7 UCSP Vila Meã, ACeS Baixo Tâmega, ARS Norte, 4605-384 Vila Meã, Portugal

**Keywords:** diet, gut microbiota, gut-lung axis, immune system, lung function, nutrition respiratory health

## Abstract

The gut microbiota is often mentioned as a “forgotten organ” or “metabolic organ”, given its profound impact on host physiology, metabolism, immune function and nutrition. A healthy diet is undoubtedly a major contributor for promoting a “good” microbial community that turns out to be crucial for a fine-tuned symbiotic relationship with the host. Both microbial-derived components and produced metabolites elicit the activation of downstream cascades capable to modulate both local and systemic immune responses. A balance between host and gut microbiota is crucial to keep a healthy intestinal barrier and an optimal immune homeostasis, thus contributing to prevent disease occurrence. How dietary habits can impact gut microbiota and, ultimately, host immunity in health and disease has been the subject of intense study, especially with regard to metabolic diseases. Only recently, these links have started to be explored in relation to lung diseases. The objective of this review is to address the current knowledge on how diet affects gut microbiota and how it acts on lung function. As the immune system seems to be the key player in the cross-talk between diet, gut microbiota and the lungs, involved immune interactions are discussed. There are key nutrients that, when present in our diet, help in gut homeostasis and lead to a healthier lifestyle, even ameliorating chronic diseases. Thus, with this review we hope to incite the scientific community interest to use diet as a valuable non-pharmacological addition to lung diseases management. First, we talk about the intestinal microbiota and interactions through the intestinal barrier for a better understanding of the following sections, which are the main focus of this article: the way diet impacts the intestinal microbiota and the immune interactions of the gut–lung axis that can explain the impact of diet, a key modifiable factor influencing the gut microbiota in several lung diseases.

## 1. The Human Gut Microbiota

The human microbiota aggregates an estimate of 100 trillion (10^14^) microbial cells that reside or colonise all body surfaces and cavities exposed to the external environment, namely, skin, eyes, the urogenital system, and the epithelial surfaces of the respiratory system and gastrointestinal tract (GIT) [1,2,3,4]. The highest microbial density, by far, is found in the GIT (gut microbiota; 3.8 × 10^13^ bacteria in the colon), where a diverse microbial community (bacteria, archaea, fungi and protists) resides, interacts with each other and the host [4,5].

In recent decades, multiomics techniques (metagenomics, metatranscriptomics, metaproteomics and metabolomics) became advanced and powerful tools capable of exploring the complex gut microbial ecosystem, namely its structure and function (Box S1 of Supplementary Material [6]. The prevalence of 1100 bacterial species has been estimated in the human collective gut microbiota, from which only about 160 such species are colonising each individual [7]. Together, gut microbiota encodes 3.3 million genes, generating an enormously diverse metabolite repertoire either from the microbial metabolism of dietary compounds or from endogenous compounds that are generated by the host and the microbes themselves (e.g., short-chain fatty acids (SCFAs), bile acid derivatives and tryptophan metabolites) [7,8,9,10,11]. Several microbial metabolites are small molecules that act as signalling molecules and substrates for metabolic reactions, deeply influencing host physiology [12]. 

A more complex and established adult-like microbiota community is reached at the age of 2–3 years [13,14]. During adulthood, the composition of the gut microbiota is relatively stable and plays a crucial role in maintaining immune and metabolic homeostasis as well as defence against pathogens [15,16]. However, it can suffer variations due to antibiotic use, lifestyle (including long-term change in diet), surgery or bacterial infections [16,17].

**Figure 1:**
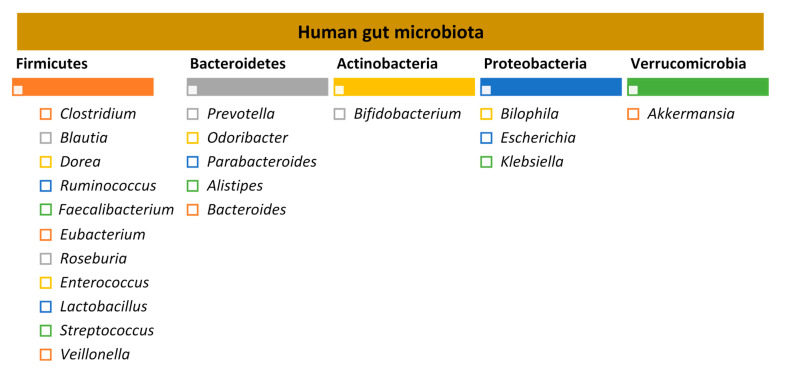
Human gut microbiota: the five major bacterial phyla and their predominant genera, according to the study by Qin et al. 2015 [22,23].

The adult human gut is predominantly colonised by two bacterial phyla, Firmicutes and Bacteroidetes, that comprise over 90% of the total gut microbial communities, followed by other subdominant phyla such as Actinobacteria, Proteobacteria and Verrucomicrobia [7,18] (Figure 1). 

The gut microbiota is considered by some authors as a “forgotten organ” or a “metabolic organ”, given its profound impact on host physiology, metabolism, immune function and nutrition [19,20,21].

## 2. Through the Intestinal Barrier: Microbiota–Immune System Interactions 

While the composition of the microbiota is deciphered, knowledge about interactions between microbes and host through the intestinal mucosal barrier is also deepened [23].

The interaction between host and both microbial antigens and microbial metabolites occurs through the single layer of epithelial cells. These cells act as guardians and transmit key signals to the host cells. These interactions influence immune responses and, ultimately, disease risk [5,10]. 

The intestinal barrier is home to presumably the largest pool of the immune cells in the human body. In fact, this is expected as this is a place to encounter a vast community of microbes, toxins, metabolites and dietary components [5,24,25]. Therefore, it is subject to a tight regulation in order to simultaneously optimise an efficient absorption of nutrients, water and electrolytes from food and provide a barrier to prevent the passage of foreign antigens, microorganisms and their toxins [26]. The gut barrier is therefore controlled by fine-tuned immune system–microbiota interactions ensuring the right discrimination between harmful and harmless antigens and resulting in adequate immune responses [5,27]. 

The innate immune system provides the intestine the ability to discriminate between pathogenic and commensal bacteria by recognising microbe-associated molecular patterns (MAMPs), such as lipopolysaccharides (LPS), peptidoglycans or flagellin, through pattern recognition receptors (PRRs) [28]. PRRs include two classes of detection molecules, the cytoplasmic NOD-like receptors (NLRs) and the membrane-bound toll-like receptors (TLRs) [29]. TLRs are mostly, but not exclusively, associated with immune and epithelial cells including macrophages, T lymphocytes and dendritic and intestinal epithelial cells [30,31].

The commensal microbiota plays a central role by reinforcing barrier immunity while protecting their own ecological niche against opportunistic pathogens [32,33]. For instance, the SCFA butyrate has protective epithelial barrier roles: colonic epithelial cells utilise microbial-derived butyrate as a source of energy; butyrate induces differentiation of colonic regulatory T cells (Tregs), which are pivotal sentinels that maintain commensal tolerance through their immunosuppressive properties, namely by expressing interleukin (IL)-10 [34,35,36]. Besides Tregs induction, important roles of the commensal microbiota have been attributed for the development of intestinal T helper 1 (Th), Th2 and Th17 cells, effector CD4^+^ T cells that secrete a wide range of cytokines (e.g., IL-17 and interferon gamma (IFNγ)), with a vital role in host defence against various types of infectious pathogens and involved in different types of tissue injury in immunologic diseases [37,38,39,40,41,42]. On the other hand, the immune system impacts the composition of the gut microbiota [5,27]. As an example, during Inflammatory Bowel Disease (IBD), chronic inflammation alters the epithelial cell tolerance to intestinal bacteria what drives changes in the gut microbiota composition [43].

Even though there is a vast density of microbial cells in the gut, it is unusual for commensal bacteria to breach the intestinal barrier, therefore avoiding tissue inflammation and microbial translocation and maintaining the mutualistic relationship between host and the gut microbiota. The homeostasis is preserved by several layers of defence against microbial translocation. In the first layer, termed “mucosal firewall”, the release of immune mediators restricts the direct contact of commensals to the epithelial cell surface and penetration, avoiding further immune activation and inflammation. The second layer of immune protection comprises the detection and killing of bacteria that penetrate the epithelium. Finally, the third layer, named “immune firewall”, reduces the exposure of commensal microbes to the systemic immune system, by inducing adaptive immune responses with the purpose to confine the bacteria in the luminal or mucosal compartments [27,32,44,45]. 

These layers include several structural and immunological constituents [32]. Besides columnar absorptive cells (enterocytes), other specialised epithelial cells, the goblet cells, are responsible for secreting mucin glycoproteins, essential components of the mucus layer [24]. The mucus layer coats the intestinal surface and protects the cells against mechanical, chemical and biological attacks. This vital role in intestinal homeostasis is evident when one looks at the constant secretion into the GIT, reaching an impressive amount of 10 L per day [46,47]. Antimicrobial peptides (e.g., Regenerating islet-derived protein III-gamma (RegIIIγ) and α-defensins) secreted by all intestinal epithelial cell lineages are mixed with the mucus and exert antimicrobial functions, preventing direct contact of the commensal microbes with the epithelium [32,47]. Additionally, immunoglobulin A (IgA) is continuously secreted by plasma cells in the lamina propria and transcytosed across the epithelium. In the lumen, the secreted IgA binds to microbes and inhibits their adherence to the epithelial surface and their penetration into the intestinal barrier [27,32]. However, some commensal microbes can breach the first barrier and reach the epithelial basolateral surface. Under normal conditions, these microbes are rapidly phagocytosed and eliminated by macrophages that reside in the subepithelial lamina propria. Notably, unlike macrophages located in other tissue sites, the intestinal macrophages do not elicit potent pro-inflammatory responses following bacterial recognition. This probably occurs to avoid tissue damage caused by pro-inflammatory (and excessive) responses to the commensal microbial community under normal conditions. Yet, pathogen species that are usually able to survive and replicate in host tissues (e.g., *Salmonella* spp.) can evade phagocytic killing by downregulating biocidal mechanisms of macrophages [27,32]. Finally, a mechanism mediated by dendritic cells and mesenteric lymph nodes restricts exposure of commensal microbes to the systemic immune system, thus limiting the induction of mucosal immunity. Dendritic cells that have sampled commensal microbes can home to the mesenteric lymph nodes and originate a local immune response but are not able to penetrate further to reach central systemic lymphoid structures (“immune firewall”) [27,44]. 

The loss of the intestinal barrier integrity facilitates translocation of deleterious components (e.g., whole bacteria, LPS, toxins, etc.) which are important players in pathogenesis of many diseases, thereby associating with various intestinal diseases, such as IBD, and systemic and metabolic disorders, like obesity or diabetes. A wide range of factors can compromise intestinal homeostasis and increase the permeability of the barrier that includes the presence of pathogens, environmental stress, high-fat/high-sugar diets, drugs and antibiotics, among others [48].

## 3. Diet and Gut Microbiota

Diet has been considered a major environmental factor affecting gut microbiota composition. Pinpointing the exact interactions between dietary components and microbiota might offer clues towards a better understanding of disease pathogenesis, enabling improved strategies for prevention and treatment [49]. The impact of single food components (macronutrients and micronutrients), salt, food additives and dietary habits (vegan, vegetarian, gluten-free, ketogenic, high sugar, low fermentable oligosaccharides, disaccharides, monosaccharides, and polyols (FODMAP), Western-type and Mediterranean diets) on gut microbiota composition has been extensively covered in a review by Rinninella et al. [50]. The multiple studies published in recent years linking dietary components or dietary patterns and gut microbiota, metabolite production and/or intestinal barrier integrity in humans are summarised in Table S1 of the Supplementary Material.

## 4. Diet, Gut Microbiota, Immune System and Lung Diseases

### 4.1. The Cross-Talk between the Gut and the Lungs: Long-Reaching Immune Modulation

Recent findings are beginning to untangle a link between gut microbiota and lung immunity, the gut–lung axis [51,52,53]. In this section, we focus on dietary components and gut microbiota that influence lung immunity and lung diseases. By way of illustration, a fibre-rich diet does not only modify the composition of both lung and gut microbiota (e.g., by altering the ratio of Firmicutes to Bacteroidetes), but also protects against allergic inflammation through increased circulating levels of SCFAs. This highlights the therapeutic potential of fibres in chronic obstructive pulmonary disease (COPD) or asthma, for example [54,55,56].

The dynamics and intimate relationship occurring in the lung-gut axis could be partially derived from the fact that both gastrointestinal and respiratory mucosal tracts share the same origin and aspects of physiology and structure, such as the direct contact to the mouth and pharynx. Both have a physical barrier with projections of microvilli (gut) or cilia (respiratory tract) that also participate in conjunction with lymphoid tissues to the local immune system. Besides this, they also share secretory IgA and mucus-producing goblet cells. Indeed, they derive from the same common embryonic organ, the foregut [53,57,58].

**Figure 2:**
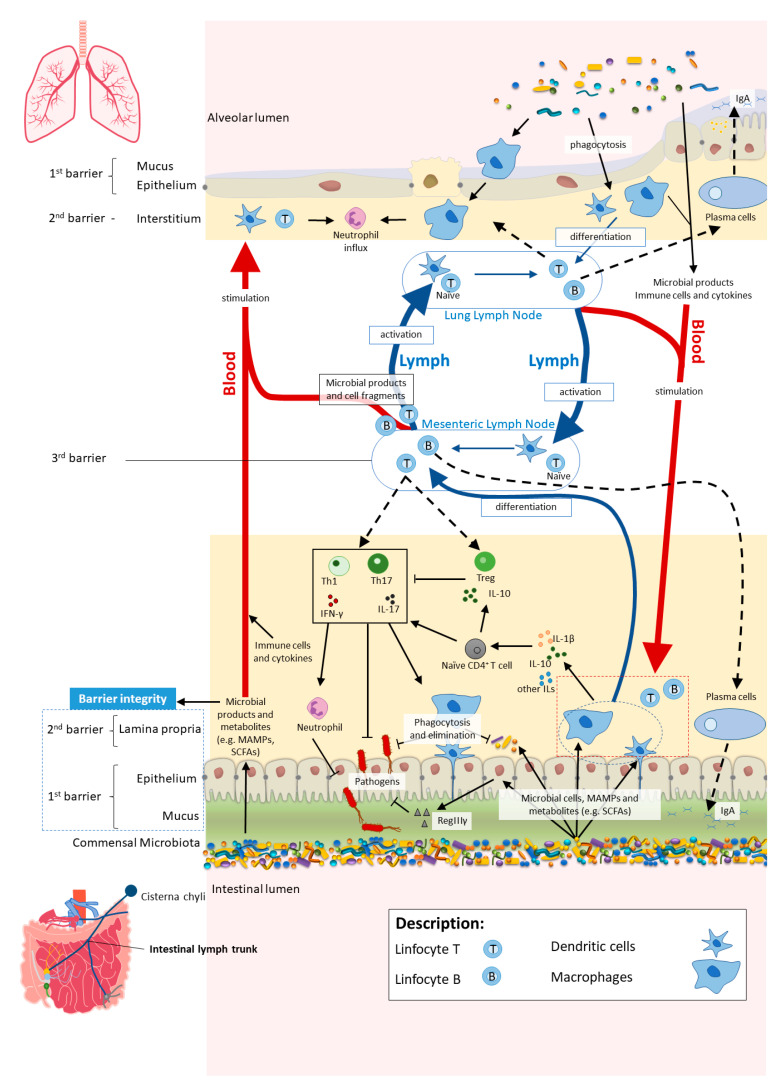
In homeostasis, microbe-associated molecular patterns (MAMPs) from the gut microbiota are recognised by pattern recognition receptors (e.g., Toll-like receptors (TLRs)) and induce antigen presenting cells (APCs), such as macrophages and dendritic cells (DC), to produce interleukins (e.g., IL- 1β and IL-10) to regulate immune responses by different subsets of T cells, neutrophils and macrophages, among others. Activated APCs induce differentiation of naïve CD4^+^ T cells into CD4^+^ regulatory T cells (Treg) (which are crucial for both maintaining tolerance to commensal microbiota and regulating other immune cells), and other effector T cells such as Th1 or Th17 (expressing cytokines, e.g., IL-17 and interferon gamma (IFNγ)) with a central role in host defence against invading pathogens, while controlling the expansion of commensals. Microbial cells or their products in the lamina propria are either phagocytosed and eliminated or transferred to mesenteric lymph nodes (MLN) by APCs, where they induce differentiation of the T and B cells. Activated B and T cells move back (black dashed arrows) to the intestinal mucosa to directly act on their target or to continue to trigger other immune cells. The majority of activated B cells differentiate into immunoglobulin A (IgA)-producing plasma cells. Bacterial metabolites, such as short-chain fatty acids (SCFAs), and expression of antimicrobial peptides (e.g., RegIIIγ) by epithelial cells (induced by TLR activation by MAMPs) reinforce the intestinal barrier integrity. Proposed pathways of the gut–lung axis that would explain the impact of the gut microbiota on the lung immunity include the migration of: (1) activated T and B cells from the MLN to distal sites such as the lung epithelium and lung lymph nodes, through lymph and blood; (2) microbial products and metabolites or surviving bacteria from the intestinal mucosa to the lung, through systemic propagation by lymph and blood circulations. Although not yet well established in the literature, the other way around has been proposed as well (from lung to gut), with the lung microbiota exerting effects in the intestinal mucosa. Scheme based on Bingula et al. [52].

The cross-talk between the gut and the lungs occurs through lymph and bloodstream circulatory systems and is crucial for passing long-reaching “immunological information” between organs [52,59,60]. There seems to be a “common mucosal response” where the effects exerted by the gut microbiota and their metabolites on the intestinal mucosal immunity influence the immune response at distal mucosal sites, such as the lungs [60,61,62]. The exact mechanisms by which the gut impacts on lung immune responses, although not fully understood, include systemic propagation of bacterial-derived components (e.g., LPS), metabolites (e.g., SCFAs) and migrating immune cells [53,60,63,64,65]. The interactions within gut–lung axis are bidirectional as the lung can also influence the gut, for instance, by lymphocyte migration and inflammatory cytokines [65] (Figure 2). 

SCFAs, mainly propionate, acetate and butyrate, resulting from the fermentation of undigested soluble dietary fibres by the gut microbiota, are important immunomodulatory metabolites and their role has been extensively explored in the gut–lung axis. SCFAs exert direct or indirect effects on the function of several cells including epithelial, innate and adaptive immune cells [63].

SCFAs mostly exert anti-inflammatory effects in the intestine by modulating epithelial and immune cell functions through the activation of G protein-coupled receptors 41, 43 and 109A (GPR41, GPR43 and GPR109A) and the inhibition of histone deacetylases [63,66]. As examples, SCFAs have a protective role in intestinal barrier cell survival and repair mechanisms, through GPR43- and GPR109A- mediated NLR family pyrin domain containing 3 (NLRP3) inflammasome activation on intestinal epithelial cells [67]. Butyrate promotes an anti-inflammatory milieu in the colon by signalling through GPR109A on colonic macrophages and dendritic cells to induce IL-10 production, an anti-inflammatory cytokine that plays a critical role in steady-state immune homeostasis [68].

The direct role of SCFAs in the lungs is most likely not substantial as circulating SCFAs do not seem to accumulate in the airways and there is no significant production of SCFAs in place. As such, it is feasible that SCFAs exert a direct effect on immune cells in the periphery with later recruitment to the lungs [54,63,69]. Recent studies have proposed other mechanisms of action for SCFAs. SCFAs influence hematopoietic precursors production (in particular, myelopoiesis) in the bone marrow to resolve airway inflammation and the maintenance of homeostasis [63]. During allergic airway inflammation, propionate produced from the microbial metabolism of high-fibre diet in mice, and in a GPR41-dependent manner, increased the generation of macrophage and dendritic cell progenitors (MDPs) and later seeding of the lungs with dendritic cells presenting high phagocytic capacity but unable to trigger Th2-mediated allergic airway inflammation [54]. In the same line, dietary supplementation with SCFAs in mice prevented exacerbation of allergic lung inflammation by vancomycin treatment. SCFAs dampened Th2 responses through direct effects in modulating the activity of T cells and dendritic cells [70]. Conversely, during influenza infection, SCFAs produced from the metabolism of high-fibre diet in mice, trigger the differentiation of MDPs into patrolling Ly6C− monocytes, reducing neutrophil recruitment to the lungs and preventing tissue immune-associated pathology during infection [69].

Other mechanisms of the long-reaching immune modulation include the intestinal microbial metabolite desaminotyrosine (DAT) and the commensal gut segmented filamentous bacteria (SFB) [64,71]. DAT, a product generated by the gut commensal *Clostridium orbiscindens* during flavonoid metabolism, augments type I IFN signalling with a consequent reduction of lung immunopathology (less airway epithelial damage and apoptosis) during influenza virus infection [72]. SFB appear to regulate pulmonary host defence by anti-microbial protein secretion, immune cell activation and recruitment [64,73]. For instance, SFB have been reported to induce CD4^+^ T cell polarisation into Th17 response in the lungs of mice, in response to pulmonary fungal infections (*Aspergillus fumigatus*). The increase of Th17 cell counts in the lungs by SFB provided antifungal immunity and was dependent on IL-1 receptor ligands, with authors suggesting that SFB may promote the systemic release of IL-1 receptor ligands [73]. Moreover, gut microbiota dysbiosis following antibiotic treatment and concomitant gut colonisation by *Candida albicans* was found to drive potent CD4^+^ T-cell-mediated allergic airway responses to *A. fumigatus*, according to Noverr et al. [74]. Further, in a model of autoimmune arthritis (in which lung complications are common), SFB led to lung autoimmunity and pathology by inducing intestinal Th17 cells recognising both an SBF epitope and self-antigen, which are then recruited to the lungs by the lung-expressed Th17 chemoattractant, C-C motif chemokine ligand 20 (CCL20) [75]. 

In addition, the migration of immune cells from the gut to the lungs as well as MAMPs seem to be other important players in the long-reaching immune interactions [64]. The immune cells group 2 innate lymphoid cells (ILC2s) migrate from the intestinal lamina propria to the lungs, where they support anti-helminth defence and tissue repair, during infection in mice [76]. The intestinal TLR activation by the gut microbiota appears to contribute to immune defence against influenza virus infection in the respiratory system. Oral-neomycin-treated mice, which shows a depletion of all culturable Gram-positive bacteria in the colon, fail to develop immune responses to respiratory influenza A virus (IgA, Th1 and cytotoxic T cell responses). Notably, a single rectal inoculation of LPS, which mimics the effect of increased levels of commensal bacteria present in the colon, was able to completely rescue the impaired immune responses involving antibody and T-cell responses, through inflammasome-dependent cytokine activation in the lungs. Further, other TLR agonists, such as unmethylated cytidine phosphate guanosine (CpG) oligodeoxynucleotides (TLR9 agonist), polyinosinic–polycytidylic acid (Poly I:C; TLR3 agonist) and, to a lesser extent, peptidoglycan (TLR2 agonist), were also able to restore immunity to influenza virus in the lung, indicating that TLR stimuli originating from distal gut bacterial products appear to be enough to support immune priming in the lung [77]. Accordingly, TLR5-mediated sensing of flagellin in the intestine has also been shown to be pivotal for immunity after the trivalent inactivated influenza vaccine (TIV), by inducing plasma cell differentiation and by stimulating lymph node macrophages to secrete plasma cells growth factors. The impaired antibody responses observed in antibiotic-treated mice could be restored by co-injection of flagellin with TIV. The way how flagellin in the gut can impact B cell response in distant sites remains unclear, however it has been suggested that vaccine-B cells are being primed in the peripheral lymph nodes by translocated flagellin itself or other downstream signalling pathways [78]. Further, gut microbiota increases bacterial clearance against *Escherichia coli* pneumonia by TLR4 signalling in mice, namely, by increasing neutrophils infiltration and the killing activity of alveolar macrophages [79]. Finally, the recognition of the gut microbial peptidoglycan via the PRR nucleotide-binding oligomerisation domain-containing protein-1 (Nod1) primes the systemic innate immune system by promoting killing by bone-marrow-derived neutrophils of two important pathogens (*Streptococcus pneumoniae* and *Staphylococcus aureus*) [80].

The interactions discussed above sustain a central role for the gut–lung axis in lung health and disease.

### 4.2. The Diet–Gut–Lung Axis in Lung Diseases: Asthma, Chronic Obstructive Pulmonary Disease and Cystic Fibrosis

Changes in gut microbiota composition triggered by diet have been documented in chronic lung diseases and respiratory acute infections. Several studies are beginning to unravel the importance of a window of opportunity during which changes in the microbiota will have an impact on long-term health.

#### 4.2.1. Asthma

Asthma is the most common noncommunicable disease among children. Worldwide, it affects more than 339 million people, being a significant public health burden [81]. Because of its major global impact, this chronic inflammatory disorder is the first to be addressed here, trying to understand if diet can be a coadjuvant factor in the management of this disease (and easily available to everyone worldwide, cost-effective and without side effects, among others, as compared to other strategies such as commercialised probiotic supplements). 

When one talks about diet and how it modulates the composition of the intestinal microbiota with inherent health consequences, namely, predisposition for allergy and asthma, we can point one of the first most striking moments in the early life of a human being: breastfeeding. Breastfeeding shapes the neonate´s gut microbiota in virtue of exposure to the milk microbiota but also to other components such as human milk oligosaccharides, secretory IgA and anti-microbial factors [82]. Breastmilk has been shown to favour the growth of bifidobacteria and lactobacilli, and alter microbiota metabolic function and, ultimately, modulate the immune system towards allergy prevention [14,82,83]. In a Canadian prospective birth cohort study involving 3296 children, it was observed that direct breastfeeding is the most protective against risk of developing asthma, when compared to formula feeding or indirect (pumped) breast milk [84]. 

Some studies highlight the importance of a window of opportunity, in the neonatal period, during which changes in the microbiota will be critical in long-term outcomes, such as predisposing subjects to develop atopy and asthma [85]. In a longitudinal Canadian study that enrolled 319 infants and analysed stools samples at 3 and 12 months, Arrieta et al. revealed that subjects at risk of asthma exhibited transient gut microbial dysbiosis during the first 100 days of life. Bacterial genera *Lachnospira*, *Veillonella*, *Faecalibacterium* and *Rothia* were significantly reduced in children at risk of asthma. Along with the reduction in bacterial taxa, reduction of faecal acetate and dysregulation of enterohepatic metabolites were observed [86]. Similarly, in a prospective birth cohort, 308 children from the USA were followed-up from 1 to 48 months after the birth. Neonates belonging to the higher risk group for atopy and asthma had lower relative abundance of some bacteria (e.g., *Bifidobacterium*, *Akkermansia* and *Faecalibacterium*) and higher relative abundance of specific fungi (*Candida* and *Rhodotorula*) and a fecal metabolome enriched for pro-inflammatory metabolites [87]. More recently, a longitudinal study of faecal microbiota and its association with allergies or asthma development was carried in Germany, involving 440 children from infancy (5, 13, 21 and 31 weeks postpartum) through school age (6 to 11 years). Although a link between microbial diversity and asthma could not be observed, an increased microbial maturity by 5 weeks of age (suggesting a dysregulated colonisation with some bacterial taxa establishing too early) was associated with a higher risk for asthma. Besides this, *Lachnobacterium*, *Lachnospira* and *Dialister* were lower abundant taxa throughout infancy among children who develop asthma [88]. Further, in a Turkish cross-sectional study, 92 children with asthma (between the ages of 3 and 8 years) had lower counts of *Akkermansia muciniphila* and *Faecalibacterium prausnitzii* compared to healthy controls. Authors suggest that both bacterial species may induce anti-inflammatory cytokine IL-10 production and prevent the secretion of pro-inflammatory cytokine IL-12 through its secreted metabolites [89].

Notably, both genera *Faecalibacterium* and *Lachnospira*, which inversely associate with asthma, are butyrate producers utilising the butyrogenic pathway (from pyruvate), which is a marker for gut commensals [90]. Additionally, *Faecalibacterium* has been shown to produce a 15 kDa protein with anti-inflammatory properties and capable not only to suppress the NF-κB pathway in intestinal epithelial cells and but also to prevent colitis in mice [91]. In addition, *Lachnospira* and, to a lesser account, *Lachnobacterium* produce acetate. Acetate has been shown to associate with a marked reduction of allergic airway disease in a human asthma mouse-model by enhancing Treg cell numbers and function [92]. 

In a birth cohort involving children from rural areas of five European countries (Austria, Finland, France, Germany and Switzerland), SCFAs were measured in stools from 301 one-year-old children. Infants with high amounts of butyrate and propionate in faeces had significantly less atopic sensitisation and were less likely to develop asthma between 3 and 6 years of age [93]. Furthermore, in a Russian cross-sectional study, 44 adult patients with bronchial asthma presented a significant decrease on the total content of fatty acids, the absolute concentrations of acetate, butyrate and propionate and the total content of isoacids in faeces compared to healthy controls [94]. 

A more westernised diet (high intake of saturated fats, sugar and processed foods and low intake of fibre) has been adopted in the past few decades coinciding with the increased prevalence of inflammatory and autoimmune diseases, including asthma [95,96].

Some studies have shed light on the role of fibre intake on promoting both a healthy gut microbiota composition and an enhanced production of SCFA, leading to amelioration of asthmatic symptoms. In line with this, other studies analysed the direct association between SCFA increment and reduction of airway inflammation in asthma.

A pilot study conducted in Australia, involving 29 adult patients with asthma, found that 4 h after the intake of a 3.5 g dose of a soluble fibre, inulin, there was a significant reduction in airway inflammation, namely, by reduction in sputum total cell count, neutrophils, macrophages, lymphocytes, IL-8 and a marker for eosinophilic inflammation, exhaled nitric oxide. Interestingly, this was accompanied by upregulation of GPR41 and GPR43 sputum cell gene expression and an improvement in lung function. Authors further suggest that fermentation of inulin results in the activation of both receptors in immune cells in the airways [55]. 

A cohort of 36 adult UK asthma-patients presented a decrease in both the gut microbial richness and the abundance of certain butyrate-producing bacteria, such as *F. prausnitzii*, when compared to healthy counterparts. This suggests that reduction of bacteria related to SCFA metabolism in asthma might contribute to the inflammatory setting in the pathogenesis of asthma [97]. 

Metabolism of a high-fat meal triggers innate immune responses via TLR activation with subsequent increase in pro-inflammatory cytokine production, as observed in an Australian study involving 37 non-obese adults with asthma and 14 obese patients with asthma. Four hours after consumption of a high-fat meal (amount of saturated fat equivalent to 21% of total energy), patients with asthma had increased airway inflammation as seen by the increase in neutrophils and TLR4 mRNA expression in sputum, when compared to asthmatic patients given a low-fat meal. The high-fat meal suppressed bronchodilator recovery in asthma as inferred by the forced expiratory volume in the first second (FEV1)/forced vital capacity (FVC) ratio. Moreover, when comparing effects from meals with high-trans or non-trans fatty acids, sputum neutrophils were significantly higher with high-trans fatty acids [98]. It is feasible that the rise in TLR4 transcript levels from this study may be a result of an augmented translocation of LPS to the systemic circulation after high-fat consumption. Indeed, other reports previously stated a high-fat diet, especially high in saturated fatty acids, associates with a reduction in gut microbiota diversity and increased intestinal barrier permeability, provoking endotoxemia and systemic inflammation [99,100,101]. 

A recent analysis integrating dietary, microbiome and plasma metabolomics into an evaluation of the intestinal metabolome in 361 3-year old-children in the USA, revealed inverse associations between asthma and intestinal polyunsaturated fatty acids (PUFAs), which are likely representing dietary PUFAs both in excess of the amount absorbed in the small intestine and non-metabolised by the gut microbiota. Intestinal PUFAs inversely correlated with the asthma-associated intestinal microbiome. Furthermore, a diet rich in fried and processed meats was linked with asthma and with asthma-associated intestinal metabolites [102]. 

#### 4.2.2. Chronic Obstructive Pulmonary Disease

Chronic obstructive pulmonary disease (COPD) is a progressive chronic lung disease estimated to affect 4–5% of the global population and to cause 2.9 million deaths annually. COPD is characterised by prolonged airway inflammation, lung tissue damage (emphysema) and airflow obstruction [56,57]. 

A UK prospective, observational cohort study involving 101 COPD patients found that instability of the lung microbiota composition was linked with disease exacerbations. The bacterial genera *Haemophilus* and *Moraxella* (Proteobacteria) were positively associated with disease severity and exacerbation events. These genera may be involved in biofilm formation and antibiotic resistance [103].

COPD associates with multiple factors such as tobacco smoking and the use of antibiotics and oral corticosteroids, which are known to cause gut dysbiosis. Therefore, besides lung microbial dysbiosis, the occurrence of gut microbial dysbiosis in COPD patients is expected [56]. Increased small intestinal permeability, assessed by urinary excretion ratio of the orally ingested sugars lactulose/L-rhamnose, has been associated with severe acute exacerbations of COPD in 17 COPD patients hospitalised in the Netherlands, with hypoxaemic respiratory failure. The possible underlying mechanisms that may account for loss of intestinal barrier integrity are hypoxic damage and/or injury by inflammatory mediators (e.g., C-reactive protein) [104]. 

In a very recent Australian study, the faecal microbiome and metabolome were found to be significantly different between COPD patients (28 subjects) and healthy controls (29 subjects). A total of 146 bacterial species diverged between the two groups. *Streptococcus* species (e.g., *S. parasanguinis* B and *S. salivarius*) were substantially higher in COPD patients, being identified as key differentiators between COPD and healthy groups. Several species, including *Streptococcus* sp000187445, *Streptococcus vestibularis* and various members of the family *Lachnospiraceae*, also associated with reduced lung function. Among the metabolites significantly diverging between COPD and healthy subjects, N-carbamoylglutamate, which has been associated with omega-3 fatty acid intake in humans, was reduced in COPD. Authors comment that further work is necessary to pinpoint whether the identified species are contributing to the airway neutrophilia and lung function decline in COPD [105]. 

A UK cohort study, with 1551 males and 1391 females, analysed the relationship between dietary patterns, lung function and COPD. The methodology included a complete spirometry and dietary data. A “prudent” dietary pattern (high consumption of fruit, vegetables, oily fish and wholemeal cereals) was correlated with protection against impaired lung function and COPD, particularly in male smokers [56]. Authors suggested that a high antioxidant content of fruit and wholemeal bread may explain the beneficial effect of a “prudent” diet on lung function and COPD. This would also explain why the dietary effects were stronger in male smokers, as these subjects had higher levels of smoking and oxidative stress. The high oily fish intake in a “prudent” diet was also suggested to be responsible for the observed beneficial effects [106]. In the same line, an American prospective cohort study, involving more than 120,000 women and men from 1984 to 2000, found that the risk of newly diagnosed COPD was one third lower in participants who ate a healthy diet (comprising high intakes of whole grains, polyunsaturated fatty acids, nuts, and omega-3 fatty acids and low intakes of red/processed meats, refined grains and sugar sweetened drinks) when compared to those who had an unhealthy diet [107].

The high intake of dietary fibre has been recently suggested to become a therapeutic intervention in COPD as a mean to reduce chronic airway inflammation by promoting a healthy gut microbiota and increased SCFA production [56]. Lately, a prospective cohort study of 35,339 Swedish women found long-term (10 years) high fibre intake (from cereal and fruit but not vegetable sources) to be linked with a 30% lower risk of COPD [108]. Accordingly, the above-mentioned Australian study reported a lower dietary fibre intake in COPD subjects, compared to healthy controls [105]. 

#### 4.2.3. Cystic Fibrosis

Cystic Fibrosis (CF) is a progressive autosomal recessive disease characterised by a dysfunction in a chloride channel, the membrane protein cystic fibrosis transmembrane conductance regulator (CFTR), resulting in thick and sticky mucus obstructing the airways, for instance. The lungs are among the multiple organs the most severely affected, with serious lung infections like *Pseudomonas* [109]. 

An Italian randomised clinical trial, enrolling 22 children with CF compared with healthy controls, found a disrupted intestinal microbiota in CF. CF patients had reduced microbial diversity. At the species level, *Eubacterium rectale* and *Faecalibacterium prausnitzii* (both butyrate producers), *Bacteroides uniformis*, *Bacteroides vulgatus*, *Bifidobacterium adolescentis*, *Bifidobacterium catenulatum* were all reduced in children with CF. Intestinal inflammation, assessed by fecal calprotectin (CLP) and rectal nitric oxide (rNO) levels, was significantly higher in CF as compared to controls. Possibly, the depletion in CF of some of the butyrate producers presented above may explain the increase in inflammation due to lower butyrate levels. Interestingly, administration of the probiotic *Lactobacillus* GG partially restored the composition of intestinal microbiota, followed by a reduction in intestinal inflammation [110]. An American study, involving 14 children with CF under 3 years old compared with healthy controls, reported a dysbiotic microbiota in CF with considerably altered capacities for lipid metabolism, including diminished capacity for overall fatty acid biosynthesis and augmented capacity for degrading anti-inflammatory SCFAs [111].

Some studies disclose possible interactions of gut–lung axis within CF context [64]. The stool microbiota composition of 21 infants with CF was followed during the first year of life and compared to healthy controls, in the USA. In contrast to the healthy infants, CF infants had no increase in their bacterial alpha diversity over the first year of life. Furthermore, the gut microbiota of infants with CF was significantly different from that of healthy controls and was associated with airway exacerbations. Across the first year of life, a significant reduction in Bacteroides and *Roseburia* and a significant increase in *Veillonella* were detected in the cohort with CF. Additionally, *in vitro* data demonstrated that apical exposure of intestinal epithelial cells to secreted products from multiple beneficial Bacteroides species reduces IL-8 production, thus suggesting a mechanism by which the gut microbiota may influence systemic signals of inflammation in CF patients and potentially impact airway disease. Considering this, authors suggest the need for CF therapeutics that direct gastrointestinal microbiota towards a healthier state [112]. In another USA study, the respiratory and intestinal microbiota were analysed in a cohort of 7 infants with CF, from birth to 21 months. There was a high degree of concordance between the bacteria in the respiratory and intestinal tracts. Over time, microbial diversity increased in both tracts. Intestinal colonisation by 7 genera (*Roseburia*, *Dorea*, *Sporacetigenium*, *Coprococcus*, *Blautia*, *Enterococcus* and *Escherichia*) presaged their appearance in the respiratory tract and their abundances in both compartments were interrelated over time. Breast milk exposure had an impact on respiratory tract as indicated by the lung bacteria profiles related with breast-feeding. Overall, these findings highlight a potential link between nutrition and gut colonisation patterns as determinants of the lung microbial development in CF infants [113]. 

Recently, the nutrient intake and adherence to dietary recommendations were assessed in 76 CF Greek children and adolescent. Despite an optimal adherence to the energy and fat recommendations for this disease, most of the subjects had a low intake of carbohydrates and fibres and revealed a poor adherence to the Mediterranean diet [114]. This implies that there is still room to improve diet quality in CF in order to improve pulmonary function [114,115].

## 5. Perspectives

Diet is a readily modifiable factor influencing the gut microbiota. Research focusing on how the gut–lung axis acts on local and systemic immunity and the subsequent effects on human health is still in its infancy. Increasing recent evidence starts to unveil the impact of both nutrition and gut microbiota in the respiratory function. A diet rich in fibres, such as the Mediterranean diet, promotes a healthy gut microbiota composition, enhancing production of SCFAs, like butyrate. The beneficial impact on gut microbiota and its metabolites improves lung function in numerous diseases, such as asthma or COPD, by reducing local and systemic inflammation. The diet itself appears to modulate directly the lung microbiota as well. Importantly, diet is one of the most readily accessible interventions for everyone to prevent and/or ameliorate a wide range of diseases. For this fact and from the accumulating scientific evidence, we conclude that diet and nutrition should not be neglected when one talks about respiratory diseases. As such, we recommend diet as a fundamental non-pharmacological addition to lung diseases management. Gathering the already existing evidence and writing specific dietary guidelines for each respiratory disease would benefit patients and reduce the global burden of disease. Nutritional advice by clinical experts should be considered in cooperation with the already in place clinical consultations (e.g., general practice, paediatrician and pulmonology consultations). 

Curiously, very recently, it has been suggested that gut microbiota dysbiosis could contribute to severity and persistency of symptoms of COVID-19 (primarily a respiratory illness caused by the severe acute respiratory syndrome coronavirus 2 (SARS-CoV-2)), possibly via modulating host immune responses [116]. Indeed, a mild or acute respiratory syndrome accompanied by the release of pro-inflammatory cytokines (IL-1β and IL-6) can occur after SARS-CoV-2 infection of the upper and lower respiratory tract. We may hypothesise that immunomodulatory factors (such as gut microbes) able to suppress these cytokines could show beneficial effects in the management of COVID-19, as seen before in other inflammatory diseases, including viral infections [117,118]. Whether diet could play a role in the extension and severity of COVID-19 remains undetermined [119]. 

Although a large number of studies have focused in recent years on the gut–lung axis and the role of dietary components, detailed mechanistic studies and clinical intervention trials with thoroughly assessed clinical outcomes are still needed to gain insight on the intricate associations going on between diet and gut and lung microbiota composition and metabolism and their influence on the immune system. Apart from metagenomics, the functional analyses by metabolomics and metatranscriptomics should be considered for accurately pinpointing the mechanisms connecting food intake to changes in human health.

Besides diet, probiotic intake and other factors to manipulate the gut microbiota, microbial products or metabolites could be possibly used in the clinic to favour a healthy state. Probiotics may be cost-effective regarding prevention and treatment of some diseases; however, since they usually fall into the regulatory category of food or dietary supplements [120], they are not affordable to everyone. Moreover, the experimental evidence to support label health claims for probiotics are usually very challenging to provide, as there are very variables to consider: specific species, strain or dose of probiotics, duration of both treatment and effects, different host response, etc. [120,121]. For instance, *Lactobacillus rhamnosus* GG has been reported to delay or reduce the occurrence of asthma in children [122,123]. These and many other studies could pave the way for new strategies in managing a wide range of respiratory diseases. Further, it has been suggested that the use of standardised doses of purified microbial components, with proven beneficial effects, could overcome the uncertainty of whether living probiotic strains are able to colonise and function in the human tract as some people appear to be more resistant to colonisation than others [124].

Besides this, other components of the gut microbiota should be explored in future studies, specifically the mycobiota and virome, which are currently largely unexplored. Their role in the gut–lung axis should be examined with the aim of finding new therapeutic strategies to improve lung diseases. 

Finally, it must be stressed that the gut–lung axis is bidirectional and, although this review has focused on the impact of the intestine on the lung, the reverse has also been demonstrated. It is now well established that chronic and acute lung disease trigger changes in the gut microbiology and physiology [125]. The lungs, previously considered to be sterile, are now known to harbour a unique microbiota. Exploring the lung ecosystem is needed in future studies to ascertain the impact of lung colonisation on host physiology [124].

## Supplementary Materials

The following are available online at https://www.mdpi.com/article/10.3390/nu13051716/s1, Box S1. Short description of different omics; Table S1. Summary of Human nutritional intervention/observational studies with different dietary components or patterns.
