# Supplementary material for: Gut Microbiota, in the Halfway between Nutrition and Lung Function
Source: Nutrients. 2021 May 19;13(5):1716. doi: 10.3390/nu13051716 (PMC8159117; doi:10.3390/nu13051716)
Supplement: Supplementary file 1 [file nutrients-13-01716-s001.zip › nutrients-1175356-supplementary.pdf]

nutrients

MDPI

Supplemental material

Box S1. Short description of different omics.

Multiomics: complex organisms, such as the human body, have multiple cells with different functions in which microbes, nutrients, contaminants, and/or other environmental factors interact, generating a multitude of signalling pathways that can vary from defence mechanisms (immune activation) to disease and cell death. Recently, multi-omics studies are encouraged to access different layers of cell information, especially in disease state, helping to understand the flow of information between layers of two or more omics. The term "-omics/-ome" designates a broad comprehensive assessment of a set of biomolecules using high-throughput and high-resolution technologies. Omics include Genomics, Epigenomics, Transcriptomics, Proteomics, Metabolomics and Metagenomics.

Genomics: one of the earliest and the most established and developed of all the omics. Genomics evolved from genetics (individual genes), and comprises the whole genome information: exons, introns, repeats, intragenic regions and promoters. This enables to map specific genetic variants that dictate different phenotypes (ex. genetic disorders that lead to disease). In 1977, Fredrik Sanger and co-workers developed the first widely used sequencing method [1], enabling the first human genome studies in 1990 [2]. DNA synthesis is randomly terminated at different lengths, fragments are separated by 1 nucleotide size difference and fluorescence is read [1]. In 2006, a new technology allowed thousands of DNA molecules to be sequenced in parallel, the Roche 454 GS FLX inaugurated the Next Generation Sequencing (NGS) [3]. Since then, many other NGS techniques have evolved to be more efficient, cheaper, quicker and high-throughput [4].

Epigenomics: the most recent of the omics. Epigenomic consists in characterizing reversible chemical changes to DNA and histones, such as DNA methylation or histone acetylation. These covalent modifications can be caused by genetic and environmental factors and are major regulators of gene transcription and cell development [5]. Modifications can be permanent and sometimes heritable, and can lead to disease [6], such as cancer [7,8] and metabolic syndrome [9–13]. NGS technologies enable epigenome assessments [14] and the first Human epigenome is underway [15,16].

Transcriptomics: consists in studying the cell RNA transcripts. At least 76% of the Human genome is transcribed into RNA, and only ≈1.2% of this RNA is protein-coding [17–19]. NGS techniques allowed to oversee the whole RNA transcription. Thus, findings were made in short and circular RNAs [20] and, recently, the role of non-coding RNA was discovered to have significant contribution in mammalian physiological processes, such as to adipose differentiation [21], and others [22].

Proteomics: is the cell protein state in a giving condition. Proteomics can be quantitative (peptide abundance) and qualitative (modifications and interactions). 2D gel techniques are the beginning of these studies, comparing whole protein profiles between two conditions [23]. With this laborious technique it was hard not only to reproduce results but also to isolate/pin-point proteins that are modified, interact with other proteins or alter quantity. Mass Spectrometry (MS) based technologies revolutionized the proteomics field allowing high-throughput and high-resolution analysis [24]. Ultra-high pressure liquid chromatography coupled with high resolution Mass Spectrometer detectors are able to analyse large protein fractions (identifying and quantifying), and measure post-translational modifications (e.g. glycosylation, ubiquitination), protein oxidation (carbonylation) and other modifications [24].

Metabolomics: this relatively recent field enables to measure the whole spectrum of small molecules, such as amino acids, fatty acids, carbohydrates, and other metabolites. Small molecules relative and targeted abundance were enabled when new technologies such as high-resolution MS and Nuclear Magnetic Resonance spectroscopy emerged [25–29]. New metabolites have been discovered to play a significant role in signalling pathways (e.g. SCFAs) [27].

Metagenomics: the study of genetic material directly from environmental samples. Microbiomics, the largest of Metagenomics, consists in identifying and quantifying microbes: bacteria, fungi and viruses. Most studies focus on bacteria (bacteriome since it was found out that the 16S rRNA gene enables to identify and phylogenetically correlate with other bacteria). Before molecular techniques, only culture-based methodologies were available and were unable to give a glance on the bacteriome. With sequencing, the microbiome field is rapidly expanding, helping to identify the microbiota and its interactions with other organisms, e.g. gut microbiota. Everyday new NGS techniques enable cheaper sequencing of full genes (e.g. whole 16S rRNA gene) and parts of full genomes. This provides additional resolution to distinguish closely related microbes and determine their function/phenotype in a given microbial community [30,31].

Table S1. Summary of Human nutritional intervention/observational studies with different dietary components or patterns.

Dietary component/ pattern			Study design (species: Human)	Impact upon the composition of gut microbiota, metabolite production and/or intestinal barrier integrity	Reference
Carbohydrates	Monosaccharides	Fructose	Observational cohort study: 52 overweight/obese participants, 23 females, 29 males (mean age 17.3 years), majority of Hispanic background. Dietary macronutrients (including fructose) evaluated using 24-h diet recalls.	High dietary fructose correlated with lower abundance of beneficial gut genera *Streptococcus* and *Eubacterium*, which are involved in carbohydrate metabolism.	[32]
			Non-randomized, crossover, interventional study: 12 metabolically healthy subjects, 7 females and 5 males (mean age 26.3 years). Nutritional standardization occurred during a 4-day period; then complex carbohydrates were either substituted by fructose or glucose (25% of total energy intake, ≈160 g/day, provided as jelly) for 3 days (a washout phase of at least 3 weeks separated these 2 replacement events).	Increased bacterial endotoxin values as well as TLR2, TLR4 and myeloid differentiation factor 88 (MYD88) mRNA expression levels were found in peripheral blood mononuclear cells. Authors suggest these effects can translate into an altered intestinal barrier function due to the ingestion of large amounts of fructose. However, they refer this needs to be further addressed in future studies.	[33]
		Lactose	Prospective, single-center, interventional study: 16 cow's milk protein-allergic (CMA) infants (mean age 7.5 months) and 12 healthy control infants (mean age 6.9 months). CMA infants successively fed with extensively hydrolyzed formula without lactose and an equal lactose-containing formula (for 2 months/diet).	The presence of dietary lactose significantly increased bifidobacteria and lactobacilli counts and decreased *Clostridia*, *Prevotella* and *Bacteroides* numbers in faecal samples of allergic children, approaching the values found in healthy controls. Finally, lactose significantly upregulated total SCFA concentration, mainly acetate and butyrate. Reported effects clearly show beneficial prebiotic potential of dietary lactose in these allergic infants.	[34]
	Oligosaccharides	Fructooligosaccharides (FOS); Galactooligosaccharides (GOS)	Prospective, randomized, double-blinded, placebo-controlled, parallel arm, interventional study: 80 healthy participants (aged 18 to 55 years) allocated to 1 out of 4 groups: FOS (2.5, 5 and 10 g/day) or maltodextrin (10 g/day (placebo)), during the dosage phase. Sample collection occurred at basal phase (day 1 to day 60), dosage phase (day 60 to day 150) and follow-up phase (day 150 to day 210).	Prebiotic intake resulted in a significant increase in gut microbiota diversity, namely the beneficial *Bifidobacterium* and *Lactobacillus*, when compared to placebo. However, withdrawal of FOS (follow-up) led to a reduction in the overall abundance levels. A positive impact of FOS was observed in certain butyrate-producing microbes, like *Ruminococcus*, *Faecalibacterium* and *Oscillospir*. The increase in butyrate-producing bacteria and bacterial diversity by FOS consumption reinforces the beneficial impact of these prebiotics upon the host.	[35]
			Randomized, placebo-controlled, interventional study: 45 healthy young individuals (aged 20 to 25 years) assigned to 1 out of 3 groups that were supplemented with *Lactobacillus salivarius* UBL S22 (probiotic strain), with or without prebiotic FOS (10 g/day), or placebo (gelatin capsules), for 6 weeks.	The probiotic alone and probiotic + FOS combination modulated the gut microbiota by significantly increasing the total counts of beneficial lactobacilli and decreasing *Escherichia coli*, when compared to baseline and placebo group. The coliform count was significantly reduced in the probiotic + FOS group, but not in the probiotic alone. Overall, the effects of the probiotic + FOS combination were more pronounced than the ones of probiotic alone. These results highlight the positive modulation of gut microbiota by probiotics and FOS consumption, by increasing commensal groups and by reducing potentially harmful bacteria.	[36]
			Randomized, double-blinded, placebo-controlled, parallel, inter-	Bifidobacteria proportion was significantly higher in the GOS+FOS treated-women *versus* the maltodex-	[37]

		ventional study: 48 healthy pregnant women (aged 18 to 45 years, in their last trimester of pregnancy) supplemented, 3 times/day, with 3 g GOS/long chain FOS (ratio 9:1) or placebo (6 g maltodextrin) from the 25th week of gestation until delivery.	trin-treated ones. However, percentages of lactobacilli did not differ between the 2 groups. In neonates, GOS and long chain FOS supplementation did not affect bifidobacteria and lactobacilli percentages, microbial diversity and foetal immunity.	
	**Inulin**	Randomized, double-blinded, placebo-controlled, parallel, interventional study: formula-fed healthy term newborns completed the first 4 months of life with control (68 infants) or 0.8 g/dL oligofructose-enriched inulin (63 infants) supplemented infant formula. Additionally, 57 breastfed newborns (BF) were included for comparison.	Oligofructose-enriched inulin supplemented formula allowed newborns to have a microbiota composition closer to that of BF newborns, with a tendency toward increased total *Bifidobacterium* counts. Additionally, supplemented formula-fed newborns had lower numbers of *Enterobacteriaceae* and *Bacteroides* and showed softer stools and higher deposition frequency compared to the control group. Notably, the latter parameters were more similar to those of the BF infants. In conclusion, oligofructose-enriched inulin supplemented formula promotes a gut microbiota closer to that of breastfeeding.	[38]
		Randomized, double-blinded, cross-over, interventional study: 54 participants (aged 20 to 75 years) with a daily consumption of 12 g inulin or 12 g maltodextrin (placebo), over a period of 4 weeks.	Inulin consumption increased *Bifidobacterium* and *Anaerostipes* abundances, while decreasing *Bilophila* genus. Interestingly, *Bilophila* population (containing known pathobionts) reduction associated with improved constipation-related quality-of-life metrics. In this regard, authors suggest inulin interventions can be taken into consideration for prebiotic research.	[39]
**Polysaccharides**	**Starch and Non-starch**	Randomized, single-blinded (investigator), controlled, crossover, interventional study: 50 participants (aged 20 to 65 years) at risk of developing metabolic syndrome, completed two 8-week dietary interventions comprising whole grain diet (179±50 g/day, rich in dietary fibre) and refined grain diet (13±10 g/day), separated by a washout phase of 6 or more weeks.	Whole grain did not significantly induce major changes in faecal microbial composition, diversity and metabolic function, although it exerted other significant effects, such as reduced systemic low-grade inflammation and body weight, when compared to refined grain. Authors mention that future studies should rather focus on studying the effect of specific grains, like oat, rye and wheat, which may exert different effects on the gut microbiota.	[40]
		Randomized, double-blinded, placebo-controlled, crossover, interventional study: 31 healthy volunteers (16 females and 15 males; aged 20 to 42 years) completed two 3-week study periods with daily consumption of 48 g breakfast cereals, either wheat bran (WB) or whole-grain (WG) cereals, separated by a 2-week washout phase.	(The WG grain endosperm is composed mainly of starch. The WG germ, a minor fraction of the grain in wheat, is made up of a complex mixture of lipids, proteins and some mainly soluble carbohydrates, while WB is composed of dietary fibres such as cellulose, hemicellulose and arabinoxylan as well as polyphenolic lignins). Numbers of faecal bifidobacteria and lactobacilli were significantly higher upon WG ingestion *versus* WB (although lactobacilli is also augmented in WB). No significant differences in faecal SCFA were reported and no adverse intestinal symptoms were disclosed. Stool frequency was increased by WB ingestion. Finally, daily WG wheat consumption induced a marked prebiotic effect on the composition of the human gut microbiota, which may add to the favorable physiological effects of WG wheat.	[41]
		Randomized, double-blinded, crossover, interventional study: healthy Chinese women and men (10+9, respectively; aged 18 to 55 years) enrolled into 2 groups. All individuals received a uniform diet from the 1-week run-in period to the end of the trial, supplemented with either high amylose resistant starch (RS; 40 g) or energy-matched control starch, for 4 weeks separated by a 4-week	At the phylum level, no differences were found for composition among groups. After RS ingestion, fifteen bacterial genera significantly decreased *versus* baseline levels (e.g., *Bacteroides*, *Anaerostipes*, *Holdemanella*, *Blautia*, *Coprococcus*_1, etc), except for *Ruminococcaceae_UCG-005* genus which showed a significantly increased abundance. Also, an increasing abundance pattern of *Akkermania* and *Victivallis* was observed after RS consumption compared to baseline. A significant increase in serum acetate value was found after RS consumption. Additional changes (see original article) were reported in the gut microbiota. In conclu-	[42]

		washout phase.	sion, changes in microbiota after RS intake are associated with the control of glucose metabolism and abdominal adiposity.	
		Randomized, crossover, interventional study: 19 adults with metabolic syndrome (median age 60 years) undertook two 4-week interventions with a healthy carbohydrate diet, enriched with arabinoxylan (AX) and resistant starch (RS; high concentration of dietary fibre), and a low-fibre Western-style diet (minimal concentration of dietary fibre).	AX and RS enriched-diet reduced total species richness of intestinal microbiota but increased bacterial community heterogeneity, both between and within subjects. Upon high-fibre diet, *Bifidobacterium* was increased whereas bacterial phyla associated with dysbiosis were reduced (Firmicutes, Bacteroidetes and Proteobacteria) as compared to the low-fibre diet. Conversely, *Bacteroides, Sutterella* and *Ruminococcus* were enriched with the low-fibre diet. SCFAs acetate and butyrate also increased with AX and RS supplementation when compared to the low-fibre diet. Isobutyrate and isovalerate (reduced protein fermentation) were decreased. AX and RS intake changes gut microbiota and SCFAs composition, with a putative positive impact upon metabolic syndrome and colonic health.	[43]
**Proteins**	**Meat-based proteins; Plant-based proteins**	Randomized, double-blinded, controlled, parallel arm, interventional study: 24 regularly endurance training men (mean age around 35 years) assigned to protein (10 g of whey isolate + 10 g of beef hydrolysate; n=12) or maltodextrin supplementation (control) for 10 weeks.	Protein supplementation resulted in a significant increase of daily protein ingestion values which resulted in a decreased abundance of health-related taxa, including *Roseburia*, *Blautia*, and *Bifidobacterium longum*, and an augmented abundance of Bacteroidetes phylum. Even though *Roseburia* and *Blautia* genera produce SCFA, changes in fecal SCFA levels were not observed, with authors claiming that the unveiled microbiota changes were not sufficient to alter SCFA production since the abundance of other SCFA producers, such as *F. prautznii*, was maintained. Species within the Bacteroidetes phylum have proteolytic activity which could explain the increased abundance of this phylum due to increased substrate availability during protein supplementation. To put it briefly, a small increase in protein ingestion reduces health-promoting bacteria.	[44]
		Randomized, 2-arm (high or low-saturated fat), crossover, interventional study: 113 heathy adult participants (aged 21 to 65 years) consumed, within each arm, three 4-week isocaloric diets (with 2-week washout periods between them) to study the effects of red meat, white meat or non-meat protein.	Chronic red meat diet, but not white or non-meat diets, increased circulating and urinary trimethylamine N-oxide (TMAO), a gut microbiota-dependent atherogenic metabolite. Isotope tracer analysis, in 13 subjects, showed red or white meat increased gut microbiota-dependent production of trimethylamine (TMA) and TMAO from carnitine but not from choline (nutrient precursors enriched in red meat) *versus* non-meat diet. Finally, dietary red meat augmented systemic TMAO (associated to atherosclerotic heart disease pathogenesis) through changes in gut microbiota, besides reducing renal TMAO excretion.	[45]
		Randomized, double-blinded, parallel arm, interventional study: 38 overweight volunteers (aged 18 to 45 years) undertook 3-week isocaloric supplementation with milk protein casein, soy protein or maltodextrin (control).	Casein and soy high–protein diets (HPDs) did not significantly change microbiota composition and diversity, but were responsible for a shift in bacterial metabolite profile. Both HPD induced a reduction in fecal butyrate *versus* the maltodextrin diet, due to high-protein and low-indigestible carbohydrate intake. Also, both HPD resulted in increased concentration of amino acid-derived bacterial metabolites, which implies a metabolic alteration toward protein degradation by the gut microbiota. Changes in rectal mucosa gene expression (related with mucosal homeostatic processes, such as cell cycle or cell death) were observed for HPD but not for the control diet. In conclusion, HPDs should be considered with some caution as they modified the luminal environment in the large intestine mainly by increasing amino acid-derived bacterial metabolite levels though reducing butyrate concentration, which may be detrimental for colonic health.	[46]

**Fat**	**Saturated fat; Unsaturated fat; Cholesterol**	Cross-sectional, observational study: 60 women in the second trimester of pregnancy, from the Norwegian NoMIC cohort. Fat ingestion assessed using food questionnaires and taxonomic differences in their gut microbiota analyzed 4 days after delivery.	Differences in fat consumption (namely, cholesterol, saturated and unsaturated fat) associated with changes in phyla composition. In detail, cholesterol and mono-unsaturated fat associated with increases in Proteobacteria (known to encompass multiple pathogens and to have pro-inflammatory properties) relative to other phyla but saturated fat related to a relative decrease in Proteobacteria. Increased intake of mono-unsaturated fat also resulted in a relative increase in Firmicutes and Bacteroidetes and a reduced Actinobacteria/Proteobacteria ratio. Increased saturated fat intake related to an increase in the Other/Proteobacteria as well as Other/Firmicutes ratios (where Other includes all remaining phyla besides Actinobacteria, Proteobacteria, Firmicutes and Bacteroidetes). In contrast, increased cholesterol ingestion resulted in an increase in Firmicutes and Proteobacteria relative to Other phyla. At the genus level, *Lachnobacterium* (higher relative abundance) and *Ruminococcus* (lower) were differentially abundant against increased saturated fat intake. *Lachnobacterium* and *Methanobrevibacter* showed higher relative abundance against increased trans-fat ingestion. In addition, microbes belonging to Actinobacteria and Firmicutes (that were not identified at the genera level) increased and decreased, respectively, against increased ingestion of poly-unsaturated fat. In conclusion, certain types of fat, such as cholesterol and mono-unsaturated fat, may be linked with a pro-inflammatory gut microbiota, potentially explaining their adverse effects on human health. The compositional change found in the maternal gut microbiota may translate into corresponding changes in infant gut microbiota, modulating infant health.	[47]
		Randomized, parallel, interventional study: 38 overweight and obese subjects (aged 18 to 65 years) assigned to consume an excess of 1000 kcal/day, for 3 weeks, of diets rich in either saturated fat, unsaturated fat or simple sugars.	Microbiota overall community structure remained highly constant during interventions but relative abundances of individual taxa were altered in a diet-dependent way: overfeeding unsaturated fat increased butyrate producers while saturated fat increased Proteobacteria. *Bilophila* genus significantly related to overfeeding-induced liver fat increase, independently of the diet arm. Finally, specific bacteria abundance exerting detrimental or protective effects on the host associated with the saturated fat and the unsaturated fat groups, respectively.	[48]
**Vitamins**	**Fat-soluble vitamins**	Cross-sectional, observational study: 60 pregnant women, from the Norwegian NoMIC cohort. Vitamins ingestion assessed using food questionnaires in the second trimester and taxonomic differences in their gut microbiota analyzed 4 days after delivery.	Higher vitamin D and retinol dietary intakes (the former presenting a stronger effect) associated with reduced microbial diversity. In summary, vitamin D and retinol associated with relative increases in Proteobacteria (known to encompass multiple pathogens and to have pro-inflammatory properties). Specifically, vitamin D consumption associated with increases in Actinobacteria/Proteobacteria, Actinobacteria/Bacteroidetes, Proteobacteria/Firmicutes, and Other/Bacteroidetes ratios (where Other includes all remaining phyla besides Actinobacteria, Proteobacteria, Firmicutes and Bacteroidetes) whereas higher retinol ingestion related to the increase in the ratio of Proteobacteria relative to Actinobacteria and Firmicutes. In contrast, higher vitamin E ingestion related to an increase in Other/Bacteroidetes ratio but to a reduction in Proteobacteria relative to Firmicutes, Actinobacteria and Other. Distinctly abundant genera against increasing intake of fat-soluble vitamins occurred: *Sutterella* (lower relative abundance) against vitamin E, *Staphylococcus* (higher) against vitamin D and *Methanobrevibacter* (higher) against retinol.	[47]

		In conclusion, the findings here diverge from studies that propose vitamin D as a protective player against some diseases, like asthma. High vitamin D (and retinol) seem to associate with adverse health effects by the relative increase in pathogenic bacterial groups, while the effects by high vitamin E ingestion are opposite to this trend.	
	Non-randomized, one group pretest posttest, interventional study: 80 healthy women (mean age 21 years). All participants were given a weekly oral dose of 50,000 IU vitamin D_3, for 12 weeks.	Vitamin D_3 supplementation (with no other major self-reported dietary change), significantly increased the average circulating 25(OH) D levels across the group (post-supplementation 25(OH) D values were 3 times higher than at baseline). Compared to baseline, vitamin D_3 supplementation (with no other major self-reported dietary change) increased both gut microbiota overall diversity and Bacteroidetes relative abundance while decreasing Firmicutes relative abundance. Authors comment these results associate with improved gut health, as described in the literature.	[49]
	Randomized, double-blinded, placebo-controlled, interventional study: 38 adults with Cystic Fibrosis (CF) completed the study: 20 classified as vitamin D insufficient + 18 subjects classified as vitamin D sufficient [aged 43 ± 17 years (mean ± SD)]. Vitamin D-insufficient subjects were randomized to receive 50,000 IU of oral vitamin D_3 (aged 34 ± 10 years; n=10) or placebo (aged 32 ± 11 years; n=10) weekly, for 12 weeks. Vitamin D-sufficient subjects were followed longitudinally for 12 weeks, not receiving any further intervention.	Gut microbiota differed significantly when the vitamin D status was taken into account. At baseline, when comparing the vitamin D-insufficient and vitamin D-sufficient groups, it was observed that taxa belonging to the class Gammaproteobacteria was significantly enriched in the former. Members of the genus *Lactococcus* were enriched after 3 months of oral vitamin D_3 treatment *versus* baseline. Also, the potentially pathogenic *Veillonella*, at the genus level, and *Erysipelotrichaceae*, at the family level, were found enriched in the vitamin D-insufficient patients receiving placebo *versus* those receiving vitamin D_3 supplementation. In CF patients, vitamin D-insufficient status associates with modifications in microbiota composition that induce inflammation; oral vitamin D_3 supplementation seems to lead to a shift toward commensal microbial communities.	[50]
**Polyphenols**	Randomized, double-blinded, placebo-controlled, interventional study: 37 overweight and obese men and women (mean age 37.8 years). During 12 weeks, individuals were supplemented with either a combination of epigallocatechin-3-gallate plus resveratrol (EGCG+RES, 282 and 80 mg/day, respectively) or partly hydrolyzed microcrystalline cellulose-filled capsules (placebo).	Men had higher abundance of Bacteroidetes than women and, at baseline, this abundance was correlated with a more pronounced EGCG+RES-induced increase in postprandial fat oxidation. EGCG+RES supplementation significantly decreased Bacteroidetes abundance and reduced *Faecalibacterium prausnitzii* in men but not in women. Other genera were not altered by the intervention with polyphenols. Fat oxidation was increased in men with polyphenols supplementation but not in women. In conclusion, the combination of both polyphenols modifies the gut microbiota leading to an augmented postprandial fat oxidation, a beneficial metabolic effect, in men but not women.	[51]
	Randomized, double-blinded, placebo-controlled, parallel arm, interventional study: 66 healthy men (aged 18 to 45 years) consumed a (poly)phenol-rich extract (116 mg, 75 g aronia berries; n=23), a whole fruit powder (12 mg, 10 g aronia berries; n=23) or maltodextrin (placebo; n=20), for 12 weeks.	Compared to placebo, consumption of aronia extract significantly increased faecal *Anaerostipes* genus abundance (butyrate producer), while consumption of aronia whole fruit powder significantly increased Bacteroides. There were no changes in microbial diversity. Correlation analysis identified significant associations between changes in vascular function (flow-mediated dilation), aronia-derived phenolic metabolites and specific gut microbial genera (e.g. *Dialister*, *Roseburia*). Authors suggest the abovementioned link could be related to the ability of gut microbes, such as *Dialister*, to release potentially bioactive phenolic compounds along with propionate and butyrate (SCFAs) that could ameliorate vascular health.	[52]
	Randomized, controlled, parallel arm, interventional study: 78	PP and LCn3 significantly increased microbial diversity and Bifidobacteria numbers, respectively.	[53]

			individuals (mean age 53-56 years across all groups) at high cardiometabolic risk allocated to consume one out of four 8-week diets (isoenergetic). Diets: (a) low long-chain n-3 polyunsaturated fatty acids (LCn3) and polyphenols (PP) (1.5g/day and 365 mg/day, respectively), (b) high LCn3 (4 g/day), (c) high PP (2903 mg/day) or (d) high LCn3 and PP (4 g/day and 2861 mg/day, respectively).	In subjects at high cardiometabolic risk, gut microbiota composition was influenced by diets naturally rich in PP or LCn3 and concomitant changes in glucose/lipid metabolism were observed. So, this study demonstrates that these diets modulate gut microbiota in association with cardiometabolic risk profile improvement.	
**Mineral water**		**Bicarbonate-rich water**	Sequential, interventional study: 19 healthy individuals, 7 men and 12 women (mean age 46.9 years). Volunteers opened only 1 bottle/day of bicarbonate-rich mineral water (BMW) or tap water (TW) and drank it 30–60 min before the 3 main meals (500 mL of BMW or TW divided before breakfast, lunch and dinner). This cycle was repeated twice. Weeks 1 and 3 and weeks 2 and 4 were consumption periods for TW and BMW, respectively.	The composition of faecal lean-inducible bacteria, family *Christensenellaceae,* increased significantly after BMW intake, as compared to TW. Also, family *Dehalobacteriaceae* abundance increased after BMW consumption and was positively correlated with *Christensenellaceae*. Authors suggest consumption of BMW might potentially prevent obesity by increasing lean-inducible bacteria, *Christensenellaceae* and *Dehalobacteriaceae*. Additionally, 19 metabolites in blood [counting metabolites related to glycolysis along with 3 amino acids (methionine, tyrosine and glycine)], were significantly different between BMW *versus* TW drinking periods.	[54]
**Food additives**	**Synthetic fibres**	**Polydextrose**	Randomized, 3-period crossover, double-blinded, placebo-controlled, interventional study: 21 healthy adult men (aged 21 to 28 years) consumed bars containing no supplemental fibre (placebo), polydextrose (21 g/day) and soluble corn fibre (SCF; 21 g/day), each for 21 days.	Polydextrose or SCF supplementation, compared to placebo, increased the relative abundance of Bacteroidetes, and decreased the relative abundance of Firmicutes, shifting the Bacteroidetes:Firmicutes ratio. Additionally, a positive correlation between total dietary fibre intake and Bacteroidetes:Firmicutes ratio was reported. However, this ratio was not related with BMI. Several taxonomic shifts would argue against a beneficial impact of SCF or polydextrose, including decreased *Dorea, Eubacterium* and *Roseburia*, which produce butyrate. Individuals consuming polydextrose and SCF had a bacterial gene composition more similar to each other than to the ones taking placebo. Carbohydrate, amino acid, lipid, cofactors and vitamins metabolism genes were shifted after each fibre supplementation. Finally, authors comment that under the current prebiotic definition umbrella, the results described above present conflicting data on the prebiotic effects of SCF and polydextrose.	[55]
	**Artificial Sweeteners**	**Sucralose, Aspartame**	Randomized, double-blinded, crossover, controlled, interventional study: 10 female and 7 male healthy participants (aged 18 to 45 years) undertook 2 interventions, each lasting 14 days and separated by a 4-week washout interval. The sweeteners consumed by each participant consisted of a standardized dose of 14% (0.425 g) and 20% (0.136 g) of the acceptable daily intake (ADI) for aspartame and sucralose, respectively.	No significant differences were found in both the median relative proportions of the most abundant bacterial taxa (genus and family) and microbiota community structure, when comparing before and after treatments with the two sweeteners. Also, SCFAs were not affected by sucralose and aspartame consumption. The doses used for sucralose and aspartame in the study resembled a consumption of approximately three 355 mL beverage cans/day, with authors suggesting that daily repeated consumption of these sweeteners in typical high consumption doses have marginal impact on gut microbiota composition or SCFA production levels.	[56]
		**Saccharin**	Randomized, parallel arm, double-blinded, placebo-controlled, interventional study: 46 healthy lean men and women (mean age 24.91 to 32.92 years across all groups) supplemented for 2 weeks with pulp filler (placebo), sodium saccharin (400 mg/day), lactisole	Fecal composition at any taxonomic level, microbial diversity or microbial activity, such as SCFA production, were not altered by pure saccharin supplementation. Also, it did not induce glucose intolerance. These findings emphasize that the suggested saccharin use is likely safe for healthy consumers that wish to replace dietary sugars for caloric control or weight management.	[57]

	(sweet taste receptors inhibitor; 670 mg/day), or saccharin with lactisole administered in capsules twice a day, aiming the maximum ADI.		
**Dietary patterns Western and Mediterranean Diets**	Randomized, crossover, controlled, parallel arm, interventional study: 10 healthy participants (18-25 years old) assigned to 4-day fast food (FF) diet (exemplifying the Western diet) and 4-day Mediterranean (Med) diet, with a 4-day washout in between.	Some fibre-fermenting bacteria in the Firmicutes phylum increased significantly after the Med diet and decreased after the FF diet (*Lachnospira*, *Roseburia*, *Butyricicocus*, etc), whereas some bile-tolerant bacteria increased after FF (*Bilophila*, *Collinsella*, *Alistipes*, etc). Indole-3-lactic acid and indole-3-propionic acid, bacterially produced metabolites shown to confer beneficial effects on neuronal cells, decreased after the FF diet and increased after the Med diet. Differences in background diet may explain the interindividual variability in response to the treatments, even though on average *Bilophila* was decreased after Med and increased after FF. In conclusion, FF diet changes the microbiome toward a profile that has been associated with chronic metabolic diseases, whereas the Med diet alters the microbiome and plasma microbial metabolites toward a healthier profile, even for a short period of each diet.	[58]
	Randomized, controlled, parallel arm, interventional study: healthy overweight and obese subjects, 43 female and 39 male (aged 43±12 years), with a habitually low intake of fruit and vegetables and a sedentary lifestyle consumed a Mediterranean diet tailored to their habitual energy intakes (Med), or maintained their usual diets (Con), for 8 weeks.	Overall microbial richness was not changed by the Med diet in comparison to ConD diet. The Med intervention, compared to Con, improved the inflammatory status of individuals experiencing an increase in gut microbiome gene richness, and led to an increase in potentially beneficial species such as the fibre-degrading *Faecalibacterium prausnitzii*, *Roseburia* and members of the *Clostridiales* and *Lachnospiraceae*taxa, linked to butyrate precursor functional pathway. A decrease in *Ruminococcus torques* and in the potentially proinflammatory *Ruminococcus gnavus* were also reported for Med diet. Individuals with the utmost reduction in fecal bile acids (Med diet arm) consistently also exhibited a relative reduction in *Bilophila wadsworthia*. The Med intervention did not significantly increase fecal SCFA concentrations, with authors speculating that a likely enhanced gut epithelial function may have raised SCFAs utilization and absorption, thus hindering the observation of their increase due to higher fibre ingestion. In conclusion, Med seems to remodel the intestinal microbiome towards a state promoting cardiovascular and metabolic health.	[59]

References for the supplemental material:

1. Sanger, F.; Nicklen, S.; Coulson, A.R. DNA sequencing with chain-terminating inhibitors. *Proc. Natl. Acad. Sci.* **1977**, *74*, 5463–5467, doi:10.1073/pnas.74.12.5463.
2. International Human Genome Sequencing Consortium Initial sequencing and analysis of the human genome. *Nature* **2001**, *412*, 565–566, doi:10.1038/35087627.
3. Green, R.E.; Krause, J.; Ptak, S.E.; Briggs, A.W.; Ronan, M.T.; Simons, J.F.; Du, L.; Egholm, M.; Rothberg, J.M.; Paunovic, M.; et al. Analysis of one million base pairs of Neanderthal DNA. *Nature* **2006**, *444*, 330–336, doi:10.1038/nature05336.
4. Logsdon, G.A.; Vollger, M.R.; Eichler, E.E. Long-read human genome sequencing and its applications. *Nat. Rev. Genet.* 2020, *21*, 597–614, doi: 10.1038/s41576-020-0236-x.
5. Piunti, A.; Shilatifard, A. Epigenetic balance of gene expression by Polycomb and COMPASS families. *Science (80-.).* **2016**, *352*, aad9780, doi:10.1126/science.aad9780.
6. Thiagalingam, S. Epigenetic memory in development and disease: Unraveling the mechanism. *Biochim. Biophys. Acta - Rev. Cancer* **2020**, *1873*, doi:10.1016/j.bbcan.2020.188349.
7. Gonzalez-Correa, C.A.; Mulett-Vásquez, E.; Miranda, D.A.; Gonzalez-Correa, C.H.; Gómez-Buitrago, P.A. The colon revisited or the key to wellness, health and disease. *Med. Hypotheses* **2017**, *108*, 133–143, doi:10.1016/j.mehy.2017.07.032.
8. Ebrahimi, V.; Soleimanian, A.; Ebrahimi, T.; Azargun, R.; Yazdani, P.; Eyvazi, S.; Tarhriz, V. Epigenetic modifications in gastric cancer: Focus on DNA methylation. *Gene* **2020**, *742*, doi:10.1016/j.gene.2020.144577.
9. Multhaup, M.L.; Seldin, M.M.; Jaffe, A.E.; Lei, X.; Kirchner, H.; Mondal, P.; Li, Y.; Rodriguez, V.; Drong, A.; Hussain, M.; et al. Mouse-human experimental epigenetic analysis unmasks dietary targets and genetic liability for diabetic phenotypes. *Cell Metab.* **2015**, *21*, 138–149, doi:10.1016/j.cmet.2014.12.014.
10. Stols-Gonçalves, D.; Tristão, L.S.; Henneman, P.; Nieuwdorp, M. Epigenetic Markers and Microbiota/Metabolite-Induced Epigenetic Modifications in the Pathogenesis of Obesity, Metabolic Syndrome, Type 2 Diabetes, and Non-alcoholic Fatty Liver Disease. *Curr. Diab. Rep.* **2019**, *19*, 1–9, doi:10.1007/s11892-019-1151-4.
11. Samblas, M.; Milagro, F.I.; Martínez, A. DNA methylation markers in obesity, metabolic syndrome, and weight loss. *Epigenetics* **2019**, *14*, 421–444, doi:10.1080/15592294.2019.1595297.
12. Lee, H.S.; Park, T. The influences of DNA methylation and epigenetic clocks, on metabolic disease, in middle-aged Koreans. *Clin. Epigenetics* **2020**, *12*, 1–10, doi:10.1186/s13148-020-00936-z.
13. Panchal, S.K.; Brown, L. DNA Methylation in Adipose Tissue and Metabolic Syndrome. *J. Clin. Med.* **2020**, *9*, 2699, doi:10.3390/jcm9092699.
14. Jones, P.A.; Issa, J.P.J.; Baylin, S. Targeting the cancer epigenome for therapy. *Nat. Rev. Genet.* **2016**, *17*, 630–641, doi:10.1038/nrg.2016.93.
15. Cell editorial team . A Cornucopia of Advances in Human Epigenomics. *Cell* **2016**, *167*, 1139, doi:10.1016/j.cell.2016.11.001.
16. Stunnenberg, H.G.; Abrignani, S.; Adams, D.; de Almeida, M.; Altucci, L.; Amin, V.; Amit, I.; Antonarakis, S.E.; Aparicio, S.; Arima, T.; et al. The International Human Epigenome Consortium: A Blueprint for Scientific Collaboration and Discovery. *Cell* **2016**, *167*, 1145–1149, doi:10.1016/j.cell.2016.11.007.
17. Dunham, I.; Kundaje, A.; Aldred, S.F.; Collins, P.J.; Davis, C.A.; Doyle, F.; Epstein, C.B.; Frietze, S.; Harrow, J.; Kaul, R.; et al. An integrated encyclopedia of DNA elements in the human genome. *Nature* **2012**, *489*, 57–74, doi:10.1038/nature11247.
18. Djebali, S.; Davis, C.A.; Merkel, A.; Dobin, A.; Lassmann, T.; Mortazavi, A.; Tanzer, A.; Lagarde, J.; Lin, W.; Schlesinger, F.; et al. Landscape of transcription in human cells. *Nature* **2012**, *489*, 101–108, doi:10.1038/nature11233.
19. Lee, H.; Zhang, Z.; Krause, H.M. Long Noncoding RNAs and Repetitive Elements: Junk or Intimate Evolutionary Partners? *Trends Genet.* **2019**, *35*, 892–902, doi:10.1016/j.tig.2019.09.006.
20. Barrett, S.P.; Salzman, J. Circular RNAs: analysis, expression and potential functions. *Development* **2016**, *143*, 1838–1847,

doi:10.1242/dev.128074.

21. Alvarez-Dominguez, J.R.; Bai, Z.; Xu, D.; Yuan, B.; Lo, K.A.; Yoon, M.J.; Lim, Y.C.; Knoll, M.; Slavov, N.; Chen, S.; et al. De Novo Reconstruction of Adipose Tissue Transcriptomes Reveals Long Non-coding RNA Regulators of Brown Adipocyte Development. *Cell Metab.* **2015**, *21*, 764–776, doi:10.1016/j.cmet.2015.04.003.
22. Ransohoff, J.D.; Wei, Y.; Khavari, P.A. The functions and unique features of long intergenic non-coding RNA. *Nat. Rev. Mol. Cell Biol.* 2018, *19*, 143–157, doi: 10.1038/nrm.2017.104.
23. O'Farrell, P.H. High resolution two-dimensional electrophoresis of proteins. *J. Biol. Chem.* **1975**, *250*, 4007–21, doi:10.1016/j.cbpa.2008.07.024.
24. Han, X.; Aslanian, A.; Yates, J.R. Mass spectrometry for proteomics. *Curr. Opin. Chem. Biol.* **2008**, *12*, 483–490, doi:10.1016/j.cbpa.2008.07.024.
25. Nagana Gowda, G.A.; Djukovic, D. Overview of mass spectrometry-based metabolomics: Opportunities and challenges. *Methods Mol. Biol.* **2014**, *1198*, 3–12, doi:10.1007/978-1-4939-1258-2_1.
26. Larive, C.K.; Barding, G.A.; Dinges, M.M. NMR spectroscopy for metabolomics and metabolic profiling. *Anal. Chem.* **2015**, *87*, 133–146, doi:10.1021/ac504075g.
27. Vernocchi, P.; Del Chierico, F.; Putignani, L. Gut microbiota profiling: Metabolomics based approach to unravel compounds affecting human health. *Front. Microbiol.* **2016**, *7*, doi:10.3389/fmicb.2016.01144.
28. Emwas, A.H.; Roy, R.; McKay, R.T.; Tenori, L.; Saccenti, E.; Nagana Gowda, G.A.; Raftery, D.; Alahmari, F.; Jaremko, L.; Jaremko, M.; et al. Nmr spectroscopy for metabolomics research. *Metabolites* **2019**, *9*, doi:10.3390/metabo9070123.
29. Manier, S.K.; Keller, A.; Schäper, J.; Meyer, M.R. Untargeted metabolomics by high resolution mass spectrometry coupled to normal and reversed phase liquid chromatography as a tool to study the in vitro biotransformation of new psychoactive substances. *Sci. Rep.* **2019**, *9*, 1–11, doi:10.1038/s41598-019-39235-w.
30. Cuscó, A.; Catozzi, C.; Viñes, J.; Sanchez, A.; Francino, O. Microbiota profiling with long amplicons using Nanopore sequencing: Full-length 16S rRNA gene and the 16S-ITS-23S of the rrn operon. *F1000Research* **2019**, *7*, doi:10.12688/f1000research.16817.2.
31. D'Andreano, S.; Cuscó, A.; Francino, O. Rapid and real-time identification of fungi up to species level with long amplicon nanopore sequencing from clinical samples. *Biol. Methods Protoc.* **2021**, *6*, doi:10.1093/biomethods/bpaa026.
32. Jones, R.B.; Alderete, T.L.; Kim, J.S.; Millstein, J.; Gilliland, F.D.; Goran, M.I. High intake of dietary fructose in overweight/obese teenagers associated with depletion of Eubacterium and Streptococcus in gut microbiome. *Gut Microbes* **2019**, *10*, 712–719, doi:10.1080/19490976.2019.1592420.
33. Nier, A.; Brandt, A.; Rajcic, D.; Bruns, T.; Bergheim, I. Short-Term Isocaloric Intake of a Fructose- but not Glucose-Rich Diet Affects Bacterial Endotoxin Concentrations and Markers of Metabolic Health in Normal Weight Healthy Subjects. *Mol. Nutr. Food Res.* **2019**, *63*, doi:10.1002/mnfr.201800868.
34. Francavilla, R.; Calasso, M.; Calace, L.; Siragusa, S.; Ndagijimana, M.; Vernocchi, P.; Brunetti, L.; Mancino, G.; Tedeschi, G.; Guerzoni, E.; et al. Effect of lactose on gut microbiota and metabolome of infants with cow's milk allergy. *Pediatr. Allergy Immunol.* **2012**, *23*, 420–427, doi:10.1111/j.1399-3038.2012.01286.x.
35. Tandon, D.; Haque, M.M.; Gote, M.; Jain, M.; Bhaduri, A.; Dubey, A.K.; Mande, S.S. A prospective randomized, double-blind, placebo-controlled, dose-response relationship study to investigate efficacy of fructo-oligosaccharides (FOS) on human gut microflora. *Sci. Rep.* **2019**, *9*, 1–15, doi:10.1038/s41598-019-41837-3.
36. Rajkumar, H.; Kumar, M.; Das, N.; Kumar, S.N.; Challa, H.R.; Nagpal, R. Effect of Probiotic Lactobacillus salivarius UBL S22 and Prebiotic Fructo-oligosaccharide on Serum Lipids, Inflammatory Markers, Insulin Sensitivity, and Gut Bacteria in Healthy Young Volunteers. *J. Cardiovasc. Pharmacol. Ther.* **2015**, *20*, 289–298, doi:10.1177/1074248414555004.
37. Shadid, R.; Haarman, M.; Knol, J.; Theis, W.; Beermann, C.; Rjosk-Dendorfer, D.; Schendel, D.J.; Koletzko, B. V.;

Krauss-Etschmann, S. Effects of galactooligosaccharide and long-chain fructooligosaccharide supplementation during pregnancy on maternal and neonatal microbiota and immunity - A randomized, double-blind, placebo-controlled study. *Am. J. Clin. Nutr.* **2007**, *86*, 1426–1437, doi:10.1093/ajcn/86.5.1426.

38. Closa-Monasterolo, R.; Gispert-Llaurado, M.; Luque, V.; Ferre, N.; Rubio-Torrents, C.; Zaragoza-Jordana, M.; Escribano, J. Safety and efficacy of inulin and oligofructose supplementation in infant formula: Results from a randomized clinical trial. *Clin. Nutr.* **2013**, *32*, 918–927, doi:10.1016/j.clnu.2013.02.009.
39. Vandeputte, D.; Falony, G.; Vieira-Silva, S.; Wang, J.; Sailer, M.; Theis, S.; Verbeke, K.; Raes, J. Prebiotic inulin-type fructans induce specific changes in the human gut microbiota. *Gut* **2017**, *66*, 1968–1974, doi:10.1136/gutjnl-2016-313271.
40. Munch Roager, H.; Vogt, J.K.; Kristensen, M.; Hansen, L.B.S.; Ibrügger, S.; Maerkedahl, R.B.; Bahl, M.I.; Lind, M.V.; Nielsen, R.L.; Frøkiaer, H.; et al. Whole grain-rich diet reduces body weight and systemic low-grade inflammation without inducing major changes of the gut microbiome: A randomised cross-over trial. *Gut* **2019**, *68*, 83–93, doi:10.1136/gutjnl-2017-314786.
41. Costabile, A.; Klinder, A.; Fava, F.; Napolitano, A.; Fogliano, V.; Leonard, C.; Gibson, G.R.; Tuohy, K.M. Whole-grain wheat breakfast cereal has a prebiotic effect on the human gut microbiota: A double-blind, placebo-controlled, crossover study. *Br. J. Nutr.* **2008**, *99*, 110–120, doi:10.1017/S0007114507793923.
42. Zhang, L.; Ouyang, Y.; Li, H.; Shen, L.; Ni, Y.; Fang, Q.; Wu, G.; Qian, L.; Xiao, Y.; Zhang, J.; et al. Metabolic phenotypes and the gut microbiota in response to dietary resistant starch type 2 in normal-weight subjects: a randomized crossover trial. *Sci. Rep.* **2019**, *9*, 1–11, doi:10.1038/s41598-018-38216-9.
43. Hald, S.; Schioldan, A.G.; Moore, M.E.; Dige, A.; Lærke, H.N.; Agnholt, J.; Knudsen, K.E.B.; Hermansen, K.; Marco, M.L.; Gregersen, S.; et al. Effects of arabinoxylan and resistant starch on intestinal microbiota and short-chain fatty acids in subjects with metabolic syndrome: A randomised crossover study. *PLoS One* **2016**, *11*, 1–18, doi:10.1371/journal.pone.0159223.
44. Moreno-Pérez, D.; Bressa, C.; Bailén, M.; Hamed-Bousdar, S.; Naclerio, F.; Carmona, M.; Pérez, M.; González-Soltero, R.; Montalvo-Lominchar, M.G.; Carabaña, C.; et al. Effect of a protein supplement on the gut microbiota of endurance athletes: A randomized, controlled, double-blind pilot study. *Nutrients* **2018**, *10*, doi:10.3390/nu10030337.
45. Wang, Z.; Bergeron, N.; Levison, B.S.; Li, X.S.; Chiu, S.; Xun, J.; Koeth, R.A.; Lin, L.; Wu, Y.; Tang, W.H.W.; et al. Impact of chronic dietary red meat, white meat, or non-meat protein on trimethylamine N-oxide metabolism and renal excretion in healthy men and women. *Eur. Heart J.* **2019**, *40*, 583–594, doi:10.1093/eurheartj/ehy799.
46. Beaumont, M.; Portune, K.J.; Steuer, N.; Lan, A.; Cerrudo, V.; Audebert, M.; Dumont, F.; Mancano, G.; Khodorova, N.; Andriamihaja, M.; et al. Quantity and source of dietary protein influence metabolite production by gut microbiota and rectal mucosa gene expression: A randomized, parallel, double-blind trial in overweight humans. *Am. J. Clin. Nutr.* **2017**, *106*, 1005–1019, doi:10.3945/ajcn.117.158816.
47. Mandal, S.; Godfrey, K.M.; McDonald, D.; Treuren, W. V.; Bjørnholt, J. V.; Midtvedt, T.; Moen, B.; Rudi, K.; Knight, R.; Brantsæter, A.L.; et al. Fat and vitamin intakes during pregnancy have stronger relations with a proinflammatory maternal microbiota than does carbohydrate intake. *Microbiome* **2016**, *4*, 1–11, doi:10.1186/s40168-016-0200-3.
48. Jian, C.; Luukkonen, P.; Sädevirta, S.; Yki-Järvinen, H.; Salonen, A. Impact of short-term overfeeding of saturated or unsaturated fat or sugars on the gut microbiota in relation to liver fat in obese and overweight adults. *Clin. Nutr.* **2021**, *40*, 207–216, doi:10.1016/j.clnu.2020.05.008.
49. Singh, P.; Rawat, A.; Alwakeel, M.; Sharif, E.; Al Khodor, S. The potential role of vitamin D supplementation as a gut microbiota modifier in healthy individuals. *Sci. Rep.* **2020**, *10*, 1–14, doi:10.1038/s41598-020-77806-4.
50. Kanhere, M.; He, J.; Chassaing, B.; Ziegler, T.R.; Alvarez, J.A.; Ivie, E.A.; Hao, L.; Hanfelt, J.; Gewirtz, A.T.; Tangpricha, V. Bolus Weekly Vitamin D3 Supplementation Impacts Gut and Airway Microbiota in Adults With Cystic Fibrosis: A Double-Blind, Randomized, Placebo-Controlled Clinical Trial. *J. Clin. Endocrinol. Metab.* **2018**, *103*, 564–574,

doi:10.1210/jc.2017-01983.

51. Most, J.; Penders, J.; Lucchesi, M.; Goossens, G.H.; Blaak, E.E. Gut microbiota composition in relation to the metabolic response to 12-week combined polyphenol supplementation in overweight men and women. *Eur. J. Clin. Nutr.* **2017**, *71*, 1040–1045, doi:10.1038/ejcn.2017.89.
52. Istas, G.; Wood, E.; Le Sayec, M.; Rawlings, C.; Yoon, J.; Dandavate, V.; Cera, D.; Rampelli, S.; Costabile, A.; Fromentin, E.; et al. Effects of aronia berry (poly)phenols on vascular function and gut microbiota: A double-blind randomized controlled trial in adult men. *Am. J. Clin. Nutr.* **2019**, *110*, 316–329, doi:10.1093/ajcn/nqz075.
53. Vetrani, C.; Maukonen, J.; Bozzetto, L.; Della Pepa, G.; Vitale, M.; Costabile, G.; Riccardi, G.; Rivellese, A.A.; Saarela, M.; Annuzzi, G. Diets naturally rich in polyphenols and/or long-chain n-3 polyunsaturated fatty acids differently affect microbiota composition in high-cardiometabolic-risk individuals. *Acta Diabetol.* **2020**, *57*, 853–860, doi:10.1007/s00592-020-01494-9.
54. Murakami, S.; Goto, Y.; Ito, K.; Hayasaka, S.; Kurihara, S.; Soga, T.; Tomita, M.; Fukuda, S. The Consumption of Bicarbonate-Rich Mineral Water Improves Glycemic Control. *Evidence-based Complement. Altern. Med.* **2015**, *2015*, doi:10.1155/2015/824395.
55. Holscher, H.D.; Gregory Caporaso, J.; Hooda, S.; Brulc, J.M.; Fahey, G.C.; Swanson, K.S. Fiber supplementation influences phylogenetic structure and functional capacity of the human intestinal microbiome: Follow-up of a randomized controlled trial. *Am. J. Clin. Nutr.* **2015**, *101*, 55–64, doi:10.3945/ajcn.114.092064.
56. Ahmad, S.Y.; Friel, J.; Mackay, D. The effects of non-nutritive artificial sweeteners, aspartame and sucralose, on the gut microbiome in healthy adults: Secondary outcomes of a randomized double-blinded crossover clinical trial. *Nutrients* **2020**, *12*, 1–16, doi:10.3390/nu12113408.
57. Serrano, J.; Smith, K.R.; Crouch, A.L.; Sharma, V.; Yi, F.; Vargova, V.; LaMoia, T.E.; Dupont, L.M.; Serna, V.; Tang, F.; et al. High-dose saccharin supplementation does not induce gut microbiota changes or glucose intolerance in healthy humans and mice. *Microbiome* **2021**, *9*, 1–18, doi:10.1186/s40168-020-00976-w.
58. Zhu, C.; Sawrey-Kubicek, L.; Beals, E.; Rhodes, C.H.; Houts, H.E.; Sacchi, R.; Zivkovic, A.M. Human gut microbiome composition and tryptophan metabolites were changed differently by fast food and Mediterranean diet in 4 days: a pilot study. *Nutr. Res.* **2020**, *77*, 62–72, doi:10.1016/j.nutres.2020.03.005.
59. Meslier, V.; Laiola, M.; Roager, H.M.; De Filippis, F.; Roume, H.; Quinquis, B.; Giacco, R.; Mennella, I.; Ferracane, R.; Pons, N.; et al. Mediterranean diet intervention in overweight and obese subjects lowers plasma cholesterol and causes changes in the gut microbiome and metabolome independently of energy intake. *Gut* **2020**, *69*, 1258–1268, doi:10.1136/gutjnl-2019-320438.
